# Aurora kinase A is a possible target of OSU-03012 to destabilize MYC family proteins

**DOI:** 10.3892/or.2014.3325

**Published:** 2014-07-11

**Authors:** ANDRES SILVA, JENNIE WANG, SARAH LOMAHAN, TUAN-ANH TRAN, LAURA GRENLIN, AKIKO SUGANAMI, YUTAKA TAMURA, NAOHIKO IKEGAKI

**Affiliations:** 1 Department of Anatomy and Cell Biology, College of Medicine, The University of Illinois at Chicago, Chicago, IL 60612, USA; 2 Department of Bioinformatics, Graduate School of Medicine, Chiba University, Chiba 260-8670, Japan

**Keywords:** neuroblastoma, protein stability, phosphorylation, protein kinase inhibitor, docking simulation

## Abstract

OSU-03012, a 3-phosphoinositide-dependent kinase-1 (PDK1) inhibitor, destabilizes MYCN and MYC proteins in neuroblastoma cells. However, AKT phosphorylation is barely detectable in neuroblastoma cells under normal culture conditions whether treated with OSU-03012 or not. This observation suggests that PDK1 is not the main target of OSU-03012 to destabilize MYC and MYCN in neuroblastoma cells. In the present study, we explored one of the possible mechanisms by which OSU-03012 destabilizes MYC and MYCN. Since Aurora kinase A is reported to phosphorylate GSK3β, leading to its inactivation, we hypothesized that one of the targets of OSU-03012 is Aurora kinase A. Comparative analysis of OSU-03012 and VX-680, a potent and specific inhibitor of Aurora kinases, showed that both inhibitors destabilized MYC and MYCN and were significantly growth suppressive to neuroblastoma cell lines. *In silico* molecular docking analysis further showed that the calculated interaction energy between Aurora kinase A and OSU-03012 was −109.901 kcal/mol, which was lower than that (−89.273 kcal/mol) between Aurora kinase A and FXG, an Aurora kinase-specific inhibitor. Finally, an *in vitro* Aurora kinase A inhibition assay using a recombinant Aurora kinase A showed that OSU-03012 significantly inhibited Aurora kinase A, although it was weaker in potency than that of VX-680. Thus, OSU-03012 has a likelihood of binding to and inhibiting Aurora kinase A *in vivo*. These results suggest that OSU-03012 affects multiple cellular targets, including Aurora kinase A, to exhibit its growth suppressive and MYC and MYCN-destabilizing effects on neuroblastoma and other cancer cells.

## Introduction

Neuroblastoma is the most common pediatric extracranial solid tumor of neural crest origin. Characteristically, it can exhibit either a favorable or an unfavorable phenotype. Favorable neuroblastomas are treatable with minimal interventions, whereas unfavorable neuroblastomas are aggressive and require extensive treatments, including autologous stem cell rescue (1). Long-term survival of children with unfavorable neuroblastoma is hence the lowest among childhood cancers. Approximately half of unfavorable neuroblastoma cases have *MYCN* amplification, which is associated with high MYCN expression, older age of onset, advanced stage disease, rapid tumor progression, and the worst prognosis (2,3). A recent study also showed that non-*MYCN* amplified neuroblastomas of the unfavorable subset express high levels of MYC instead of MYCN, which appears to be the determining factor of their aggressiveness (4).

**Figure 1:**
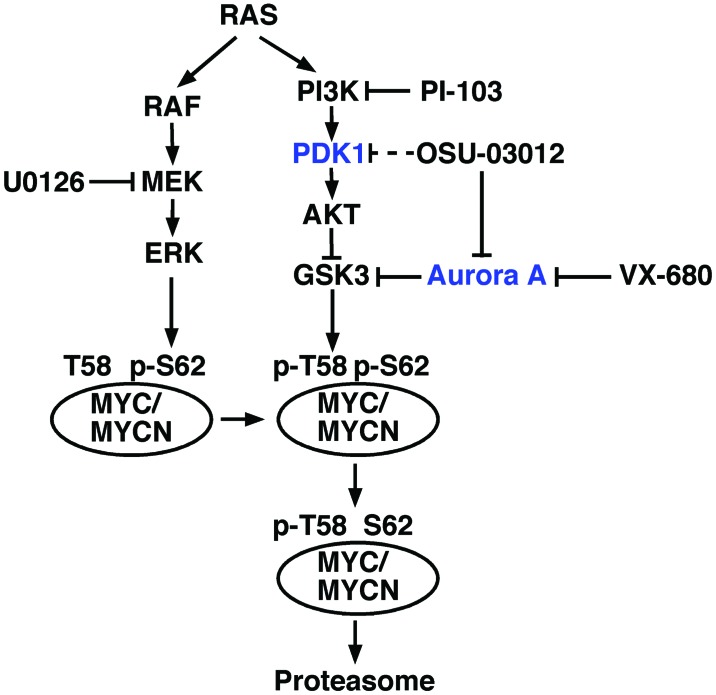
A model of the regulation of MYC family protein stability by the RAS, RAF/MEK/ERK and PI3K/PDK1/AKT/GSK3 pathways. Inhibition of RAF/MEK/ERK and/or PI3K/PDK1/AKT pathways by small-molecule inhibitors [e.g., U0126 (10) and PI-103 (21)] results in the destabilization of MYC and MYCN. Inhibition of the RAF/MEK/ERK pathway prevents phosphorylation of S62, which otherwise stabilizes MYC family proteins. S62 phosphorylation is a prerequisite for T58 phosphorylation. On the other hand, inhibition of the PI3K/PDK1/AKT pathway augments the phosphorylation of T58 by maintaining GSK3 activity, which then facilitates the degradation of MYC family proteins through proteasome proteolysis action (7–9). OSU-03012 inhibits Aurora kinase A, but not PDK1, leading to the activation of GSK3. This in turn enhances MYC family protein degradation. Alternatively, OSU-03012 binding to Aurora A causes a structural change to Aurora kinase A. This then results in the dissociation of Aurora kinase A from the MYC or MYCN protein, making them more susceptible to proteasome-mediated degradation (22).

OSU-0312 is a celecoxib-derived PDK1 inhibitor that has a growth suppressive effect on various cancer cell lines (5,6). When RAS is activated through external stimuli or mutation, two distinct cellular pathways are activated: the RAF/MEK/ERK and PI3/PDK1/AKT/GSK3 pathways. These pathways in turn affect the stability of MYC family proteins (7–9) (Fig. 1). In various cancer cell lines, when MEK is inhibited by U0126, there is a marked reduction in MYC protein expression levels with concomitant growth suppression (10). Based on these observations, it is plausible that inhibition of any point in the RAF/MEK/ERK and/or PI3K/PDK1/AKT pathways can destabilize MYC and MYCN. Hence, OSU-0312 was predicted to destabilize MYC and MYCN protein in neuroblastoma cells.

However, since PDK1 phosphorylates AKT to activate it, and OSU-03012 is an inhibitor of PDK1, it was puzzling that under normal cell culture conditions, AKT was barely phosphorylated and OSU-03012 did not affect the p-AKT status in neuroblastoma cells (as confirmed below). This observation suggests that PDK1 is not the main target of OSU-03012 in neuroblastoma cells. In addition, it has been reported that GSK3β is phosphorylated by Aurora kinase A, and that Aurora A knockdown results in reduced MYC levels (11). These observations collectively suggest that OSU-03012 inhibits Aurora kinase A, which in turn destabilizes MYC and MYCN in neuroblastoma cells (Fig. 1). In the present study, we investigated this possibility.

## Materials and methods

### Neuroblastoma cell lines

The neuroblastoma cell lines were grown in RPMI-1640 supplemented with 5% (v/v) fetal bovine serum and OPI (1 mM oxaloacetate, 0.45 mM pyruvate, 0.2 unit/ml insulin, at final concentrations). These cell lines tested negative for mycoplasma, and their identity was validated by the original source or by microsatellite analysis (P.S. White, Children’s Hospital of Philadelphia, Philadelphia, PA, USA; unpublished data). The IMR5 (a clone of IMR32) and CHP134 cell lines were obtained from Dr Roger H. Kennett (Wheaton College, Wheaton, IL, USA). The SKNBE(2)C cell line was from Robert Ross (Fordham University, New York, NY, USA). The SKNAS cell line was from Dr C. Patrick Reynolds (The Texas Tech University Health Sciences Center, Lubbock, TX, USA). CHP134, IMR5 and SKBBE(2)C are *MYCN*-amplified cell lines, whereas SKNAS is a non-*MYCN*-amplified cell line.

### MTS assay

An MTS [3-(4,5-dimethylthiazol-2-yl)-5-(3-carboxymethoxyphenyl)-2-(4-sulfophenyl)-2H-tetrazolium, inner salt] assay (a water-soluble form of the MTT assay) was performed as described in our previous study (12). OSU-03012 was purchased from Cayman Chemical Company (Ann Arbor, MI, USA). VX-680 was purchased from LC Laboratories (Woburn, MA, USA). The stock solutions of the inhibitors were prepared at 10 mM in DMSO, and stored at −20°C.

### Western blot analysis

Western blotting was performed as previously described (13,14). Light emission signals were captured by an LAS-3000 digital image analyzer (Fujifilm). Cell extracts were made in 2D gel sample buffer (9 M urea, 2% Nonidet-P40, 2% 2-mercaptoethanol and 0.32% pH 3.0 to 10.0 2D Pharmalyte), and the protein content of the samples was determined by the Bio-Rad protein assay kit using bovine serum albumin as a standard and the sample buffer as the blank. Antibodies used to detect proteins of interest are described in the figure legends.

### Molecular docking analysis in silico

The 3D structure of AURKA (PDB ID: 3DAJ) was obtained from the Brookhaven Protein Databank. The structures of FXG and OSU-03012 were constructed using MOE (version 2007; Chemical Computing Group, Montreal, Canada). The docking simulations and interaction energy calculations were performed using MOE Dock of MOE. The most stable docking structures of FXG and OSU-03012 with AURKA were displayed by MOE.

### Cell-free Aurora kinase A assay

The HTScan^®^ Aurora A kinase assay kit (Cell Signaling Technology Inc., Danvers, MA, USA) was used to examine the inhibitory activity of OSU-03012 and VX-680 against recombinant Aurora kinase A, according to the manufacturer’s instructions.

## Results

### OSU-03012 and VX-680 suppress the growth of neuroblastoma cells

**Figure 2:**
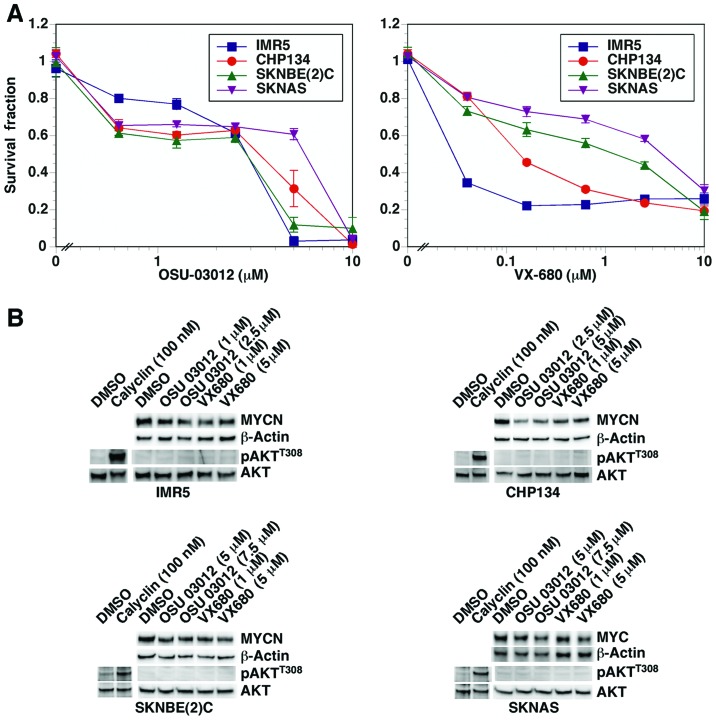
(A) OSU-03012 and VX-680 suppress the growth of neuroblastoma cells. CHP134, IMR5, SKNBE(2)C and SKNAS neuroblastoma cells were treated with OSU-03012 and VX-680 for 2 days and then subjected to MTS assay. (B) OSU-03012 and VX-680 destabilized MYC and MYCN, while AKT phosphorylation was not affected. CHP134, IMR5, SKNBE(2)C and SKNAS neuroblastoma cells were treated with OSU-03012 and VX-680 for 1 day and subjected to western blot assay. Anti-pan MYC monoclonal antibody, NCM II 143, was used to detect MYC and MYCN proteins. The rabbit anti-AKT (polyclonal) and anti-pAKT^T308^ (monoclonal, clone D25E6) antibodies were from Cell Signaling Technology Inc. (Danvers, MA, USA). Calyculin-treated (for 30 min) CHP134, IMR5, SKNBE(2)C and SKNAS cells were used to show that the anti-pAKT^T308^ could detect endogenous levels of pAKT^T308^.

First, we examined the dose response of OSU-03012 and VX-680, a potent Aurora kinase inhibitor (16), on the growth of neuroblastoma cell lines (with and without *MYCN* amplification). As shown in Fig. 2A, both compounds were significantly growth inhibitory against the neuroblastoma cells in dose-dependent manners.

### OSU-03012 and VX-680 destabilize MYC and MYCN

We next assessed the effect of OSU-03012 and VX-680 on the stability of MYC and MYCN in the neuroblastoma cell lines. As shown in Fig 2B, both compounds destabilized MYC and MYCN at low μM concentrations following one day of drug treatment.

### OSU-03012 and VX-680 do not affect the phosphorylation status of AKT

The status of AKT phosphorylation and the effect of OSU-03012 and VX-680 on it were examined next. As shown in Fig. 2B, under the normal culture condition, AKT was not phosphorylated at detectable levels nor was its phosphorylation status affected following drug treatment. The anti-pAKT^T308^ antibody used was functional, as it detected pAKT^T308^ upon treatment of the neuroblastoma cell lines with a protein phosphatase inhibitor, calyculin. This observation suggests that PDK1 is not the main target of OSU-03012 in neuroblastoma cells.

### In silico analysis reveals that OSU-03012 inhibits Aurora kinase A

**Figure 3:**
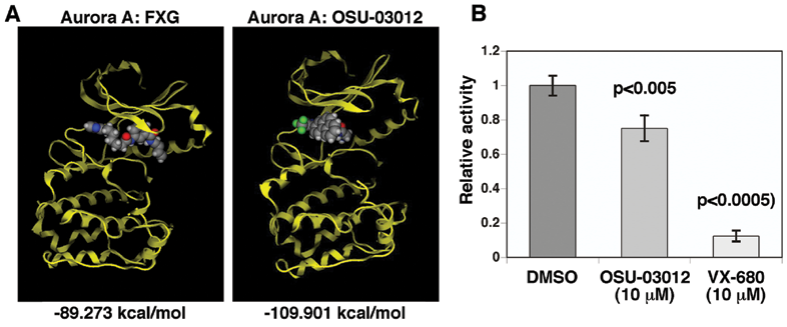
(A) Docking simulation of OSU-03012 with Aurora kinase A. The docking simulations and interaction energy calculations were performed by MOE Dock of MOE. The resulting most stable docking structures between Aurora kinase A and FXG (a positive control) or OSU-03012 were displayed by MOE. (B) OSU-03012 inhibited Aurora A kinase activity *in vitro*. Aurora A kinase inhibitory activity of OSU-03012 was assessed by the HTScan^®^ Aurora A kinase assay kit (Cell Signaling Technology) according to the manufacturer’s instructions. VX-680, a potent and specific Aurora kinase inhibitor was used as a positive control.

FXG is a derivative of compound 6, an Aurora kinase inhibitor, discovered through site-specific dynamic combinatorial chemistry by Cancilla *et al* (15). Furthermore, co-crystallization of FXG and Aurora A has been performed and the coordinates have been deposited in the Brookhaven Protein Database (PDB ID: 3DAJ). Therefore, we used this complex (FXG docking into Aurora A) as the positive control to examine whether OSU-03012 has any likelihood of binding to Aurora kinase A. We performed *in silico* docking analysis of OSU-03012 and Aurora kinase A. As shown in Fig. 3A, OSU-03012 exhibited a lower calculated binding energy in comparison to a positive control compound (FXG) against Aurora kinase A. This result suggests that OSU-03012 has enough potential to bind to and exhibit an inhibitory effect on Aurora kinase A.

### OSU-03012 inhibits Aurora kinase A in an in vitro assay

To further assess if, in fact, OSU-03012 can inhibit Aurora kinase A, we performed an *in vitro* Aurora kinase A inhibition assay. As shown in Fig. 3B, OSU-03012 inhibited Aurora kinase A, although its efficacy was less than that of the potent Aurora kinase A inhibitor, VX-680 (16).

## Discussion

It has been well documented that the stability of MYC family proteins are in part regulated through their phosphorylation status. ERK-mediated serine 62 (S62) phosphorylation appears to be the first signal regulating the MYC protein stability, which stabilizes MYC proteins. However, S62 phosphorylation triggers phosphorylation of MYC family proteins at threonine 58 (T58) via GKS3. This leads to dephosphorylation of S62. MYC proteins phosphorylated at T58 are then degraded through the proteasome (Fig. 1) (7,9).

As shown in Fig. 1, inhibition at any point in the RAF/MEK/ERK and PI3K/PDK1/AKT/GSK3 pathways by small-molecule inhibitors would therefore destabilize MYC family proteins. We initially became interested in OSU-03012 as this small-molecule inhibitor was reported to inhibit PDK1. OSU-03012 is a derivative of celecoxib, which has a weak PDK1 inhibitory activity (6). Based on this observation, Zhu *et al* developed OSU-03012 as a more potent PDK1 inhibitor through structural optimization of celecoxib.

In fact, treatment of neuroblastoma cells with OSU-03012 resulted in destabilization of MYC family proteins (Fig. 2). However, AKT, the downstream effector of PDK1, was barely phosphorylated under normal cell culture conditions, and OSU-03012 did not affect the status of AKT phosphorylation. Thus, PDK1 is not a likely target of OSU-03012, and this compound may affect another point of the signaling cascades that regulate the stability of MYC family proteins. Yacoub *et al* (17) reported a similar observation that OSU-03012 activity was not closely correlated with inhibition of PDK-1 and the phosphorylation status of AKT, and argued that OSU-03012 must have additional targets apart from PDK-1 in its cytotoxic actions in lung cancer cells. Thus, the potential target of OSU-03012, apart from PDK1, that could affect the stability of MYC family proteins remained unknown.

Notably, it was reported that knockdown of Aurora kinase A by RNAi considerably reduced the expression of MYC (11). This observation and the findings mentioned above collectively indicated that OSU-03012 could inhibit Aurora kinase A, hence reducing the stability of MYC family proteins. In the present study, we investigated this possibility. In fact, *in silico* docking analysis and an *in vitro* Aurora kinase A inhibition assay demonstrated that OSU-03012 can bind and inhibit Aurora kinase A.

To date, many small-molecule inhibitors of protein kinases have been reported (18). These inhibitors often have potencies of nM IC_50_ values against cognate protein kinases in cell-free assay (19), and they are considered highly selective. For example, VX-608 has an IC_50_ value of 36 nM against Aurora kinase A in cell-free assay (20). However, Bain *et al* (19) demonstrated that all of the small-molecule protein kinase inhibitors that they tested had substantial off-target effects on protein kinases other than their cognate kinases. Collectively, our results suggest that OSU-03012 affects multiple cellular targets, including Aurora kinase A, to exhibit its growth suppressive and MYC and MYCN-destabilizing effects in neuroblastoma and other cancer cells.
